# Perceptions of mental health providers of the barriers and facilitators of using and engaging youth in digital mental-health-enabled measurement based care

**DOI:** 10.1177/20552076241253093

**Published:** 2024-05-07

**Authors:** E.M. Bassi, K.S. Bright, L.G. Norman, K. Pintson, S. Daniel, S. Sidhu, J. Gondziola, J. Bradley, M. Fersovitch, L. Stamp, K. Moskovic, H.M. LaMonica, F. Iorfino, T. Gaskell, S. Tomlinson, D.W. Johnson, G. Dimitropoulos

**Affiliations:** 1 Faculty of Social Work, 2129 University of Calgary, Calgary, Alberta, Canada; 2 School of Nursing and Midwifery, Faculty of Health, Community, and Education, Mount Royal University, Calgary, Alberta, Canada; 3 Heroes in Mind, Advocacy, and Research Consortium (HiMARC), Faculty of Rehabilitation Medicine, College of Health Science, University of Alberta, Edmonton, Alberta, Canada; 4 Faculty of Nursing, 2129 University of Calgary, Calgary, Alberta, Canada; 5 Provincial Addiction and Mental Health, 3146 Alberta Health Services, Calgary, Alberta, Canada; 6 Brain and Mind Centre, 4334 The University of Sydney, Sydney, Australia; 7 Departments of Pediatrics, Cumming School of Medicine, 2129 University of Calgary, Calgary, Alberta, Canada; 8 Emergency Medicine, Cumming School of Medicine, 2129 University of Calgary, Calgary, Alberta, Canada; 9 Physiology and Pharmacology, Cumming School of Medicine, 2129 University of Calgary, Calgary, Alberta, Canada; 10 Maternal Newborn Child and Youth Strategic Clinical Network, 3146 Alberta Health Services, Calgary, Alberta, Canada; 11 Calgary Eating Disorders Program, 3146 Alberta Health Services, Calgary, Alberta, Canada; 12 Mathison Centre for Mental Health Research and Education, 2129 University of Calgary, Calgary, Alberta, Canada; 13 157744 Alberta Children's Hospital Research Institute, 2129 University of Calgary, Calgary, Alberta, Canada

**Keywords:** Digital mental health, measurement-based care, qualitative methods, implementation science

## Abstract

**Objectives:**

Increased rates of mental health disorders and substance use among youth and young adults have increased globally, furthering the strain on an already burdened mental health system. Digital solutions have been proposed as a potential option for the provision of timely mental health services for youth, with little research exploring mental health professional views about using such innovative tools. In Alberta, Canada, we are evaluating the implementation and integration of a digital mental health (dMH) platform into existing service pathways. Within this paper we seek to explore mental health professionals’ perceptions of the barriers and facilitators that may influence their utilization of digital MH-enabled measurement-based care (MBC) with the youth who access their services.

**Methods:**

A qualitative, descriptive methodology was used to inductively generate themes from focus groups conducted with mental health professionals from specialized mental health services and primary care networks in Alberta.

**Results:**

As mental health professionals considered the barriers and facilitators of using dMH with youth, they referenced individual and family barriers and facilitators to consider. Providers highlighted perceived barriers, including: *first,* cultural stigma, family apprehension about mental health care, and parental access to dMH and MBC as deterrents to providers adopting digital platforms in routine care; *second,* perceptions of increased responsibility and liability for youth in crisis; *third,* perception that some psychiatric and neurodevelopmental disorders in youth are not amenable to dMH; *fourth,* professionals contemplated youth readiness to engage with dMH-enabled MBC. Participants also highlighted pertinent facilitators to dMH use, noting: *first*, the suitability of dMH for youth with mild mental health concerns; *second,* youth motivated to report their changes in mental health symptoms; and *lastly,* youth proficiency and preference for dMH options.

**Conclusions:**

By identifying professionals’ perceptions of barriers and facilitators for youth users, we may better understand how to address misconceptions about who is eligible and appropriate for dMH through training and education.

## Introduction

The provision of mental healthcare services, along with the prevention of emerging mental health symptoms and the promotion of positive mental health and wellness, are globally recognized priorities outlined by the World Health Organization.^1^ These priorities acknowledge the significant burden of mental illness, characterized by high mortality and morbidity rates,^2^ thus emphasizing the critical need for effective interventions and support systems.^3,4^ Emerging evidence suggests that a substantial portion of mental health issues originate in childhood or adolescence, highlighting the importance of early intervention and support to facilitate positive long-term outcomes and mitigate the burden of mental illness in adulthood.^5,6^

In Canada, there has been a concerning increase in the prevalence of mental health and addiction concerns across all age groups (15 to 65 and over), with youth aged 15–24 exhibiting the highest rates.^7,8^ This alarming trend underscores the urgent need for comprehensive support and tailored intervention strategies to address the escalating burden faced by youth. Despite the substantial number of youth affected by mental health problems, access to appropriate treatment remains disappointingly low, with less than 20% receiving the necessary interventions.^9,10^ The high prevalence of mental health disorders among youth is accompanied by a considerable treatment gap,^11^ and even those who manage to access services often receive limited evidence-based treatment.^12^ Access to mental health services for youth is hindered by various barriers, including limited availability,^13,14^ lack of culturally responsive treatments,^14,15^ high costs,^15,16^ long wait times,^14,17,18^ geographical challenges,^15^ fragmented and siloed care,^18–21^ as well as pervasive stigma and discrimination.^15,16,18,22^ These barriers contribute to delays in accessing care and perpetuate inequalities, particularly among marginalized groups, including Black, Indigenous, and People of Color (BIPOC),^15,23,24^ as well as gender diverse populations.^25–28^

Digital mental health (dMH) presents viable solutions to support the mental health of the population.^29^ Enhancing the measurement, treatment, and monitoring of mental health services among youth can be accomplished through the utilization of dMH which offers a promising potential to address the obstacles that young people encounter when navigating the mental health care system.^30^ Studies investigating the use of mental health services in youth populations show that digital technologies can serve as a useful supplement to in-person services,^29,31,32^ with many young people open to utilizing online resources for mental health guidance, services, and support, both before and after accessing the mental healthcare system.^31,33^ dMH has the capability to address deficiencies in conventional mental health treatment by offering evidence-based interventions on a large scale,^34^ thereby making mental health support more accessible and adaptable.^35^ This potential outlined could help reduce the barriers associated with seeking mental health support among youth.

Advancements in technology have facilitated the practical and effective implementation of approaches such as measurement-based care (MBC) to enhance treatment outcomes.^36^ MBC is an evidence-based and collaborative approach in mental health care that involves utilizing patient-reported progress and outcome measures to inform treatment decisions.^37^ This approach is grounded in the principle that clinical decision-making should be guided by patient data and emphasizes the importance of actively involving patients in the care process.^38,39^ MBC is considered a fundamental component of evidence-based psychosocial treatments, as highlighted by clinical practice guidelines referenced by many professional organizations.^40^ Despite the recommendation of these guidelines by professional organizations, concerns have been raised by healthcare professionals regarding the feasibility and perceived burden associated with implementing MBC in practice.^41–43^ However, emerging evidence suggests that MBC can significantly enhance clinical outcomes by promoting timely responses to patient concerns and improving treatment satisfaction.^42,44–46^ Mental health professionals who adopt MBC may experience a heightened sense of agency in their interventions, as they can personalize treatment to meet each patient's unique needs and monitor their progress over time.^47^ Moreover, leveraging patient-reported outcome measures establishes a feedback mechanism that allows clinicians to continuously update their understanding of the patient's condition,^43^ and adjust their treatment strategies accordingly.^37,45^ Continuous measurement of patient-reported outcomes is particularly beneficial for individuals who deviate from the expected trajectory of progress.^48^

While studies have explored professionals’ perspectives on implementing MBC initiatives in mental health service settings to some extent, research concerning mental health professional views on which individuals they consider suitable candidates for dMH including MBC has not been conducted. The Electronic Mental Health for Youth and Young Adults in Alberta Project is evaluating the implementation of Innowell, a dMH platform designed to support mental health care, within existing service pathways to improve outcomes for youth with mental health and substance use problems. This study makes notable contributions to the gaps in the literature by investigating mental health professionals’ perceptions of the barriers and facilitators that may influence their utilization of dMH-enabled MBC with the youth who seek their services.

## Methods

A detailed description of the methods and analysis has been previously outlined.^49^ The University of Calgary's Research Ethics Board provided this study with approval, Project Name “Pre-Implementation and Implementation Phase of E-Mental Health for Youth and Young Adults in Alberta,” Ethics ID: REB20-1137.^49^ Participants were recruited using purposive sampling to ensure participants were invited from sites who agreed to implement the Innowell platform.^49^ Participants were invited to participate via email and during meetings where practice leads from the implementation team working within the sites in each community disseminated recruitment materials with senior decision makers and clinical supervisors in each service setting.^49^ The inclusion criteria for participants included working as a mental health provider frontline staff, management/leadership, or administrator at one of the identified sites; be proficient in written and spoken English; and lastly, use web-based technologies on a laptop/computer, tablet, or smartphone.^49^

The theoretical domains framework (TDF) was used to guide the development of a semi-structured interview guide and data analysis. The TDF provides an approach to understanding factors influencing behavioral change.^50^ A semi-structured interview guide allowed for a balance between structure and flexibility for interviewers to explore responses in more depth or to pursue unexpected avenues that were service setting specific. Each focus group included an implementation practice lead working with the implementation sites, two facilitators, and a note-taker who was often a youth research partner.^49^

This study held seven focus groups with specialized mental health services (SMHS) clinics and five focus groups with Primary Care Networks (PCNs) from eight communities across Alberta. Focus groups were conducted online via Zoom Video Conferencing software, audio recorded, and subsequently transcribed verbatim.^49^ Each focus group lasted approximately 90 minutes in length and had three to 20 participants, depending on site size and availability of mental health staff per mental health setting. Informed consent of participants and a survey of sociodemographic information were completed online before each focus group.^49^ Participants in the focus groups included mental health care professionals, administrators, and managers of SMHS and PCNs in Alberta, specifically those who serve youth with mental health or substance use concerns. Participants represented both urban and rural settings across the province of Alberta. A description of the sample can be seen below in Table 1.

**Table 1.** Descriptive information about participants (Adapted from Dimitropoulos et al. 2024)^49^

Variable name	*Descriptive*		
	*N*	*N*	%
Total participants	103		
Sex			
Female		74	71.8%
Male		16	15.5%
Missing		13	12.6%
Profession			
Counsellor/therapist/social worker		50	48.5%
Psychologist		13	12.6%
Administrator		5	4.8%
Physician or nurse		22	21.3%
Missing		13	12.6%
Age			
20–29 years		9	8.7%
30–39 years		31	30.0%
40–49 years		32	31.0%
50 and older		18	17.4%
Missing		13	12.6%
Length of time practicing			
1 to 5 years		21	20.3%
6 to 10 years		14	13.5%
11 years or more		56	54.3%
Missing		12	11.6%
Length of time at organization			
Less than 1 year		8	7.7%
1 to 5 years		44	42.7%
6 to 10 years		17	16.5%
11 years or more		22	21.3%
Missing		12	11.6%
Ethnicity^a^			
Other North American origins		20	
European origins		55	
Asian origins		10	
Other^b^		13	
Missing		17	
Level of education			
Completed college or trades school		13	12.6%
Bachelor's degree from a university		28	27.1%
Graduate school		49	47.5%
Missing^c^		13	12.6%

^a^Number does not equal the total because participants were allowed to select multiple responses. Therefore, no percentage is included for these numbers.

^b^ Other is combined sociodemographic information of less than five participants to protect anonymity. ^c^Missing includes not reported and preference to not answer.

This study follows a qualitative descriptive design.^51–53^ This methodological approach encourages the analysis to stay close to the data by describing participants’ experiences and perspectives while embracing the language they use.^54^ After each focus group, a rapid summary of the main points generated in each focus group was provided in writing to the participants within 48 hours. We asked each participant to review and provide any feedback if their thoughts, perspectives, and experiences were not captured accurately as a form of member checking. In addition to this, we shared the overall themes with our project advisory group which consisted of researchers, decision makers, and managers of the communities involved in the project to discuss and consider the findings in the context of their knowledge as experts. The qualitative analysis of the focus group data followed the six stages of thematic analysis including familiarization with the data, generating codes, constructing themes, revising themes, defining themes, and producing the report.^55,56^ The steps to the analysis were conducted iteratively, and a reflexive process was developed by moving back and forth between the six steps.^57,58^ Two young adult researchers first familiarized themselves with the transcripts. After familiarization, they independently generated preliminary codes of the data under the support and supervision from the Qualitative Lead. They subsequently exchanged transcripts and reviewed the coding to address discrepancies through consensus and ensure validation of the codes. The coders and research team (including the coders) then worked together to generate themes based on the codes. The research team participated in group discussions about the codes and the themes generated and actively generated written memos to ensure that the process was recorded and rigorous. Following these six steps in the analysis helped ensure accuracy and credibility. Our sample of 12 focus groups with 103 participants was justified through “information power,” noting that we had gathered substantial information from these groups to draw conclusions about the data.^59^ The quotes illustrated in the results have been deidentified and replaced with focus group number and participant number.

### Innowell platform

The Innowell platform is a customizable dMH tool that was co-designed alongside health professionals and individuals with lived experience.^60–62^ The platform embraces a personalized approach and is designed to deliver MBC of 20 different Mental Health and Wellness measures.^61^ The measures provide data about the service user, assist with management of mental health challenges, and support with monitoring treatment to support personalized clinical treatment.^61^ On average, it takes approximately 40 minutes for the youth to complete the initial full question set inclusive of all 20 domains. Thereafter, they can update specific domains that are a priority for them. The Addiction and Mental Health domains include: 1. Psychological Distress; 2. Suicidal Thoughts and Behaviors; 3. Psychosis-like Experiences; 4. Mania-like Experiences; 5. Social and Occupational Function; 6. Self-harm; 7. Tobacco Use; 8. Alcohol Use; 9. Social Connectedness; 10. Depressed Mood; 11. Anxiety; 12. Physical Health; 13. Sleep–wake Cycle; 14. Post-traumatic Stress; 15. Eating Behaviors and Body Image; 16. Cannabis Use; 17. Grief and Loss; 18. Spiritual Health; 19. Cultural Connectedness; and lastly, 20. Resilience. In addition, the platform offers apps and e-tools for each health domain and includes options about what may be available in the community and ways to inform the mental health care professional about what the youth would like to explore, discuss, and potentially prioritize. The platform is intended to be applied within mental health service settings and used collaboratively as a tool between mental health care professionals and youth to assess and monitor mental health symptoms and make decisions about what level of care is needed.^59^ While the platform could be used among many populations, the Electronic Mental Health For Youth and Young Adults in Alberta Project was designed to assess feasibility with youth aged 15 to 24. Innowell is the platform used to assist clinicians in judgment about treatment courses using MBC as a contributor for treatment strategies. For this study, Innowell is a representative of dMH-enabled MBC more broadly.

## Results

### Summary

Each theme explores the perspectives of participants regarding barriers and facilitators related to youth accessing and using dMH-enabled MBC in specialized mental health services and primary care settings. A summary of these facilitators and barriers is shown in Table 2, and each theme is delineated in greater detail below.

**Table 2.** Summary of themes and supporting codes.

	Themes and sub-themes
Barriers to youth access and use of digital mental health	Cultural stigma, family apprehension about mental health care, and parental access to dMH and MBC are deterrents to providers adopting digital platforms in routine care Issues of consent and access to information
	Are we equipped to deal with mental health crisis triggered by the youth using dMH?: Perceptions of increased responsibility and liability for a youth in crisis *Responsibility and liability*
	Perception that some psychiatric and neurodevelopmental disorders in youth are not amenable to dMH
	Youth readiness to engage with dMH and MBC
Facilitators to youth access and use of digital mental health	Youth with mild mental health concerns are the best fit for dMH!
	Youth motivated to report dynamic change in mental health
	Youth proficiency and preferences for dMH and MBC

## Barriers

### Theme 1: cultural stigma, family apprehension about mental health care, and parental access to dMH and MBC are deterrents to providers adopting digital platforms in routine care

#### Issues of consent and access to information

Participants in both SMHS and PCN settings identified parents or supportive adults’ (hereinafter referred to as “parents”) involvement and access to their child's activities on a dMH platform as an impediment to their adolescent clients agreeing to use dMH. For example, the legal requirement to obtain consent for minors (<18 years of age) was viewed as a barrier to offering dMH to some youth who may be reluctant to disclose their mental health concerns to their parents. One PCN participant succinctly echoed this concern: “*… I unfortunately work with some parents that don’t think that mental health is a real thing and so they’re really against the idea of any sort of counselling or even professional involvement”* (FG4_P3). Participants uniformly agreed that the potential of parents accessing their child's information in dMH would be unacceptable to most youth and would dissuade them from using such a platform if suggested by their provider. One participant from a PCN encapsulated this concern by sharing: “*…I can just think of a couple parents right now who would want to have access to seeing the assessments and things like that and a lot of kids might have some apprehension around that”* (FG4_P4). Participants concurred that parental access to personal details about mental health and responses on assessment measures embedded within dMH may deter youths’ use, and therefore professionals’ adoption of dMH in their routine clinical interactions with their clients.

Participants across SMHS and PCN settings similarly argued that they do not have the training, time, and resources to work with parents on how best to support their youth using dMH to manage their mental health. SMHS participants were concerned about their lack of capacity to respond to parents who may become alarmed by their child's MBC outcomes. One participant shared: “*…are parents going to be monitoring this [the dMH platform]? Because then, now we’re going to field calls from family members saying, ‘hey, my kid is escalating and what are you doing about it?’”* (FG6_P1). Participants understood that youth may add their parents as a “supportive other” to the dMH platform while limiting what access they can view. However, questions persisted about parents becoming concerned and anxious about their child's MBC results, leading to requests to meet with professionals to receive information and resources and elicit support from a professional on how best to intervene to help their child when this may not even be necessary.

Participants in both settings cited family and cultural stigma as prohibiting many youth from seeking professional support for their mental health including accessing dMH as supplementary intervention. For example, one SMHS participant stated that “*…there may be more stigma in terms of reaching out for help in some cultures”* (FG16_P6). Moreover, some participants suggested that newcomer or undocumented families may experience distrust of professionals, leading them not to permit their child to use mental health services, including dMH. One SMHS participant shared: “*…a lot of temporary workers, undocumented families coming in for mental health support. I would feel like you know, is that something[dMH]… in their context, they would feel comfortable utilising?”* (FG7_P1). Overall, participants reported that some parents and their children may fear personal repercussions including judgments from their extended family and community and therefore avoid dMH and mental health services altogether.

### Theme 2: are we equipped to deal with mental health crisis triggered by youth using dmh?: perceptions of increased responsibility and liability for a youth in crisis

A majority of participants raised concerns about lacking the support and resources to rapidly respond to youth in acute crises using dMH. Of particular concern was the possibility of a youth being identified on the platform as scoring high for suicidal thoughts and behaviors outside of regular sessions and work hours. In one PCN focus group, participants feared missing the notifications about such clients because a majority of their mental health professionals worked part-time (FG2). Full-time PCN professionals in other focus groups also shared concerns that they may not have the capacity to respond in a timely manner: “*[Say] I’m back-to-back this afternoon, what happens if I get a flag that one of my patients is suicidal? Where does that fit in my day?”* (FG4_P6). Many participants felt that a high demand for youth mental health services, combined with a limited number of mental health care professionals, creates a situation where they fear being unable to respond to urgent youth mental health concerns. The introduction of dMH in their services was perceived as exacerbating an already untenable mental health crisis in the community.

Participants in both settings further noted capacity concerns in relation to youth who may be recurrently identified as scoring high for suicidal thoughts and behaviors on the dMH platform. While a small number of participants did view crisis support as their primary responsibility, most mental health professionals specified that their programs are “*not a crisis service”* (FG11_P1) and are intended to provide non-urgent care to youth with mild to moderate acuity. Youth requiring crisis care while using dMH would therefore need to access such resources through another professional or service (e.g. emergency department, crisis line). Despite crisis support being out of their scope of practice, participants stated that they treat youth who experience persistent suicidal thoughts and behaviors: “*I have some patients who I've seen for literally a year and if you question them, they will always come up suicidal”* (FG2_P2). Participants anticipated that the use of dMH with these youth would add considerable strain on their time due to being under-resourced and already carrying a huge workload as mental health professionals. For example, one SMHS participant shared that:

“[…] we have some high-risk clients that have some chronic suicidal thoughts. So, from the perspective of a therapist, you know, that might be concerning like, oh my god, now do I need to find time in my day – this is going to give us more information which can be good but also more responsibility to track these kids down when actually there's a safety plan in place […] So, it just might place a bit of a burden on the clinician.” (FG12_P1).

#### Responsibility and liability

In addition to concerns around professional capacity, participants in both settings voiced apprehension about their responsibility and liability for youth identified as scoring high for suicidal thoughts and behaviors. One SMHS participant shared: “*When she was mentioning that we would be alerted if someone rated high suicide, my first [thought] was, well, what am I supposed to do with that then? […] Well, hopefully they reached out and dealt with the crisis services or followed through on our safety plan”* (FG11_P1). Many of the participants advocated for the dMH platform not to be used in their services as an avenue for youth to access “*crisis interventions,”* because of providers “*fears”* regarding risk and responsibility (FG13_P1). This illustrates that many professionals view crisis intervention as both outside their scope of practice and as adding a level of responsibility and liability that some are uncomfortable with or feel they cannot meet with their current caseload.

### Theme 3: perception that some psychiatric and neurodevelopmental disorders in youth are not amenable to dMH

Participants across both settings raised concerns that some youth with specific types of psychiatric diagnoses may be unable to appropriately use the dMH platform. Both SMHS and PCN participants worried that exaggerated mental health responses on MBC in the dMH platform may inappropriately contribute to more youth requiring intensive services such as inpatient or day treatment. One SMHS participant speculated that youth may oversubscribe to certain symptoms because “*…they haven’t figured out adaptive ways of getting their needs met […] …it's a way of communicating how much pain and suffering they’re in”* (FG12_P1). Conversely, participants were concerned that some youth may be unable to self-assess their mental health status, symptoms, and level of functioning using the MBC features objectively and accurately in dMH: “*…I wonder also about things like reporter bias […] when self-assessments are being used. I think with very many youth you’re dealing with a broad spectrum of cognitive, emotional, and psychological health insight of awareness. So, that puts a couple questions in there for me about the validity of self-reporting”* (FG23_P4). Although participants expressed significant concerns about exaggeration or inaccurate or over-reporting of mental health symptoms, no participants in any of the focus groups identified under-reporting or minimizing of distress as a barrier (nor facilitator) to implementing dMH.

In both settings, participants again questioned the suitability of dMH for youth living with enduring and persistent mental health conditions, such as bipolar disorder, schizophrenia, substance use disorders (SUDs), and personality disorders. One participant shared, “*I think it's a great platform, but would it work for say, our chronic […] our longer term bipolar or schizophrenic, mental health people? I see this working very efficiently for anxiety”* (FG6_P1). Some SMHS participants felt that youth with SUDs may be reluctant to engage in dMH tasks, such as the ongoing completion of measures, due to ambivalence about changing their relationships to drugs and/or alcohol. Concerns were raised regarding organizational capacity: “*My other concern would be if they’re identified and need more therapeutic intervention by a clinician and we have already given them their 3 to 6 sessions, what is going to happen when we get an influx? […] Some of our personality disorders will inflate their scores to get back to the one-on-one therapy. Because we know that that's what the majority of people prefer”* (FG2 _P3). Another PCN participant reiterated concerns with professionals’ responsibility and liability: “*…I’m talking mostly about your personality disorders […] it's going to flag them every week and what if we don’t reach out to them every week. I just worry frankly about some of the liability aspects of that”* (FG2_P2). Given that most participants were overburdened in terms of their caseload, there exists a concern that professionals and their organizations may be unable to provide adequate assistance to youth with certain types of disorders who may be identified on the dMH platform as requiring greater and urgent support. 

Irrespective of a youth's diagnosis, participants anticipated that providing youth with access to dMH-enabled MBC may inadvertently encourage youth to draw erroneous conclusions about their symptoms. The potential for youth to self-diagnose through the completion of measures was noted by some participants as part of a larger “trend” which emerged during the COVID-19 pandemic. For example, one participant described obtaining a psychiatric diagnosis as desirable for youth and alluded that this may create additional responsibilities for professionals:

“…since this online schooling… recently, it's a trend among the teenagers like they still want to have a diagnosis, like mental health kind of diagnosis. And they talk about this openly with their friends and with their classmates. And I know a lot of times kids come to us and [say] ‘I want a diagnosis; do you think I have this? I have this, I have that?’ I’m just wondering, it's a concern again that if you’re giving them like all this bunch of questionnaires to, you know, to fill up and then… […] I’m worried that they would kind of misuse it in a way that, you know, they will fill up a form and then they would show it to their friends and, ‘hey, look, my anxiety is so high’, and then we will also be getting these messages…” (FG12_P3)

Other participants shared similar concerns that the online and accessible nature of MBC results in the dMH platform may allow for ease of data-sharing and self-diagnosis among youth and their peers. For one participant, concerns with self-diagnosis were related to the possibility for MBC to exacerbate specific mental health symptoms and worsen a youth's mental state. This was considered a significant deterrent to their implementation of dMH: “*…and what if we allow them to enter a platform like let's say on anxiety, and if it starts to catastrophize the symptoms or how they feel, right… […] I’m just thinking about some of the youth I’ve worked with and some of the clients I have, I have to be really careful as a clinician what I did let them access because it could get almost worse in a way”* (FG23_P2).

Participants in both SMHS and PCN settings also considered specific neurodevelopmental disorders and disabilities as posing unique challenges for some youth using the dMH platform. For example, SMHS participants felt that youth with attention-deficit/hyperactivity disorder (ADHD) may lack the concentration required to complete the measures: “*ADHD is one of the most common issues in our clinics […] and when you have ADHD kids that are impulsive, disorganised, and inattentive they aren’t going to come back to it”* (FG12_P1). Consequently, participants advocated that youth with ADHD receive additional assistance from mental health professionals to complete measures in-session and reminders to use the platform between appointments.

Other SMHS and PCN participants perceived that youth with autism spectrum disorder (ASD) and fetal alcohol spectrum disorders (FASDs) would be unable to use dMH without experiencing significant challenges. In these examples, participants were primarily concerned with the youths’ cognitive and overall functioning. One SMHS participant shared, “*…a lot of the kids that I’ve seen historically with FASD I think would struggle with something like this […]. Like a lot of the complex diagnosis, multiple diagnosis and how that's affecting their functioning”* (FG_P2).

Finally, participants in both settings were hesitant to implement the platform for youth with learning disabilities. Several participants felt that these youth may have trouble comprehending the MBC questions by themselves. For example, one PCN participant stated that “*…we have a lot of youth that struggle with learning disabilities that might struggle with navigating the platform”* (FG2_P5). One SMHS participant questioned how to support these youth: “*… I mean we can say someone is developmentally […] is fifteen but they’re functioning at like a grade three level. So, what do we do about that…”* (FG23_P2). This quote describes many participants’ simultaneous hesitancy and desire to help the youth with learning disabilities to use MBC in dMH.

In this theme, we addressed participants’ hesitation regarding youth with specific psychiatric and neurodevelopmental disorders and their ability to engage with dMH-enabled MBC safely and effectively. In the following theme, we will discuss concerns with youth readiness to engage with dMH as a final individual barrier to dMH as noted by participants.

### Theme 4: youth readiness to engage with dMH and MBC

Where the youth is in their individual recovery journey can be a factor in professionals’ willingness to introduce and recommend the dMH platform to their client. Participants noted that some youth may have low levels of motivation and therefore may feel less inclined to dedicate time and effort to use apps and e-tools and complete measures to assess changes in their mental health symptoms and status. Participants had doubts that such youth would be interested in engaging with MBC and embedded care options as they had already observed an unwillingness to engage with therapeutic “*homework*” (FG11_P5) outside of sessions. Some professionals had also previously encountered youth who had strong preferences, positive or negative, regarding scales and measures: “*I have lots of clients who like doing some standardized tests, or things like that really freak them out and they don’t like that and that increases their anxiety”* (FG4_P4). Of particular concern was the length of the MBC protocol embedded in the dMH platform. For example, one SMHS participant described: “*[…] about how long is this going to take a youth to complete? […] I was asked to complete a survey or evaluation for a whole program – by fifteen minutes I was like, “This is too long.” […] Like I think about the time how motivated are the youth going to be if it takes too long to go through all these cards and whatnot”* (FG7_P1). Some participants had already observed reluctance by youth to complete short Internet-delivered screening measures: “*With our screening we do send them through email, and it is just one link that they need to click to access it. So, it is pretty easy. The fact that we don’t really have too much of a success rate with getting clients to do it before the appointment, I'm not too sure how it would kind of work with this platform as well”* (FG2_P6). These sentiments highlight the professionals’ previous experience with youth interest and motivation in MBC influencing their readiness to adopt dMH in their work with youth.

## Facilitators

### Theme 1: youth with mild mental health concerns are the best fit for dMH!

Throughout all of the focus groups, participants proposed that dMH should be used primarily with youth presenting with mild mental health symptoms. Participants considered the dMH platform as a “*first line of defence for low-risk youth”* (FG13_P1). Another participant recommended that professionals “*use it [the dMH platform] for our less high acuity people and […] have our therapists focus [instead] on those that truly need to see them”* (FG2_P2). They anticipated that the apps and e-tools available through the dMH platform may lessen workload burdens for professionals by reducing their time spent with the youth with mild mental health concerns.

### Theme 2: youth motivated to report dynamic change in mental health

Participants viewed youth in distress who are motivated to recover from their mental health challenges as the best candidates for dMH because of their compliance in completing measures. These participants expressed optimism that continuous completion of MBC questions embedded in the dMH platform may aid professionals to identify and provide support to their clients experiencing fluctuations in their mental health symptoms. For example, one participant perceived value in the additional information about the changing mental health status of youth gained through MBC and suggested that it may support professionals to provide early interventions:

“I think then we get to the point where we are able to assess a potential crisis before it happens and we're able to maybe deal with things quicker rather than they’re waiting and waiting and waiting for their appointment to come up, all the time they're getting worse and worse and worse and finally a crisis happens. Whereas [with] this we can gain that information as we go and as they start to decline and maybe intervene earlier rather than later” (FG2_P1).

In a different focus group, one participant pointed to the potential for youth to alert their mental health professional of a sudden deterioration in their mental health via dMH. They shared that:

“…let's say that the youth was low-risk. That they’ve accessed resources, and everything has been fine but then all of a sudden, that there's a triggering event or something changes and then the provider all of a sudden, we find out later on that they had already accessed this [the platform], this information was there and there could have been information along the way that we could have intervened sooner. But without having access to the information, we wouldn’t, we wouldn’t know that” (FG1_P1).

These quotes highlight that while many participants were apprehensive to use dMH with youth in distress, some participants felt positive about youths’ ability to share updated information about their mental health status with professionals irrespective of their acuity level.

### Theme 3: youth proficiency and preferences for dMH and MBC

Participants in both SMHS and PCN settings considered youth as well suited for dMH since they are technologically skilled and comfortable with using apps and e-tools. One PCN participant suggested that “*…most youth are pretty tech savvy, and they know way more than I do about tech…”* (FG19_P3). Many participants argued that some youth prefer using apps and e-tools to access their mental health support and therefore are the ideal clients for dMH. For example, one SMHS participant noted: “*…with that age group is, you know, a lot of these youth connect better electronically. So, I think being able to just do this in the comfort of their own home. …it feels more natural for them, maybe for some of them, to be able to share this on the device. …I think for that age group especially it's that convenient and it just feels more of a natural flow for them”* (FG12_P2). Another SMHS participant expected that some youth would be motivated to use dMH to access psychoeducational information about mental health issues and skills for coping: “*…I think there's definitely a clientele that would work really well with it. […] Anxiety Canada could refer people to go and do the modules on there and just get psychoeducation from that […]. A portion of my clients will definitely go and do that”* (FG16_P7). These sentiments among both SMHS and PCN participants draw attention to youth preferences for dMH.

## Discussion

The findings of this research provide novel insights about mental health professionals’ thoughts and opinions about barriers and facilitators pertaining to the use of dMH, including embedded MBC, apps, and e-tools. The findings suggest that the clinical decision to utilize dMH with diverse youth is complex and based on a multitude of individual and family level factors that may facilitate or hinder the use of dMH with youth. At the end of the discussion, we provide a table for recommendations for dMH implementation (See Table 3 below).

**Table 3.** Recommendations for addressing barriers and facilitators to dMH implementation.

Theme	Recommendation
Barrier 1: Cultural stigma, family apprehension about mental health care, and parental access to dMH and MBC are deterrents to providers adopting digital platforms in routine care	Ensure dMH education and training includes the extent of parent access to clinical information and if applicable, the need for parent resources to aid in understanding of what dMH is, and the benefits, risks, and features of the platform. Provide mental health professionals with training to assist in obtaining consent from parents and addressing how to promote developmentally appropriate sharing of information with parents that is directed by the youth using dMH and MBC. Provide opportunities for educational development and training for mental health professionals around family involvement in exploring dMH as a tool for their child to use to aid in exploring mental health treatment options. Strategies for effective communication, transparency, respect for confidentiality, and collaboration are key elements in approaching parent requests and expectations for information about their child's questionnaire data. Upskill and educate mental health professionals with respect to enhancing their skills and confidence in engaging diverse families when recommending new dMH options. Training could include strategies for supporting families presenting with diverse roles, relationships, and dynamics.
Barrier 2: Are we equipped to deal with mental health crisis triggered by youth using dMH?: Perceptions of increased responsibility and liability for a youth in crisis	Ensure mental health professionals understand crisis resources and features of dMH platforms, and the scope of their responsibilities to the youth in imminent crisis by ensuring knowledge of their organization's crisis protocol. To support the implementation of dMH in routine clinical care, decision makers may consider establishing crisis protocol with their clinical teams to increase uptake and sustainability. Provide comprehensive training to mental health professionals on how to use dMH with youth presenting with different levels of acuity, including mild symptoms, fluctuating symptoms, and when requiring urgent care.
Barrier 3: Perception that some psychiatric and neurodevelopmental disorders in youth are not amenable to dMH and Barrier 4: Youth readiness to engagement with dMH and MBC	Provide comprehensive training for mental health professionals to equip them with the knowledge, skills, and tools necessary to facilitate implementation and adoption of dMH with diverse clients in their service setting. Provide training to providers to assess the unique family context and the potential risks and benefits of dMH for every youth. We recommend that mental health professionals embrace a shared clinical decision-making approach, where they explore the best dMH option that is suitable in a collaborative manner with the youth. Encourage mental health professionals to empower youth to make their own decisions about using dMH after informed consent.

The first theme that emerged in our study calls attention to mental health providers’ concerns regarding the legal requirements to consent minors for treatment and the implications of parents potentially gaining access to MBC data in dMH. There is increasing literature on the role that parental consent can pose as a barrier to mental health professionals using and youth accessing dMH tools.^63,64^ Factors that may contribute to a reluctance to obtain parental consent among youth include the importance of their personal privacy, feeling that parents lack awareness and understanding of mental health, and apprehension about negative parental reactions.^64^ Other factors described within our results include the significance of family and cultural stigma, noted cross-culturally. Our findings support the literature describing the impact of stigma on youth access to dMH, especially among migrating or immigrant youth and their families.^65^ Pervasive stigma and fears of being judged by others can have an impact on youth seeking help for their mental health in general,^66^ and our findings extend this consideration to dMH tool access as perceived by mental health professionals as well. Mental health professionals require additional training on how to use dMH platforms like Innowell to determine how results from MBC are shared with youth and families that are de-stigmatizing, supporting, and empowering for youth.

The concept of mental health acuity or crisis was also significantly discussed by participants in our study. Participants frequently raised concerns about youth who experience chronic suicidal thoughts and behaviors or those in an acute crisis using the platform. Participants referenced these concerns based on their overburdened caseloads and about potential liability for not responding rapidly to a youth who scores high for suicidal thoughts and behaviors. Despite this, there is clear need and use for technology to help improve provider and service capacity for responding to suicidality.^62,67^ Participants also viewed youth with less severe mental health challenges as ideal for onboarding onto dMH and using apps and e-tools. The findings from our study are congruent with other research that show mental health care professionals prefer less responsibility for addressing imminent mental health crises^67,68^ and lack confidence in responding to high-risk behaviors.^69,70^ While professionals are concerned about the burden of workload and liability in the face of high mental health acuity, existing literature does showcase the effectiveness of dMH-enabled MBC as supportive for monitoring acuity levels, which could assist in ensuring mental health professionals are able to offer interventions when youth need them most.^71^ Innowell specifically provides region specific crisis resources, as well as access to a suite of vetted apps and e-tools, some of which are specifically designed as crisis resources. The results of our study further suggest that there is a need to train and support mental health professionals to use dMH and MBC with youth experiencing moderate to severe mental health symptoms and acute crises.^62^

A key concern among mental health professionals in our study is that some psychiatric diagnoses, such as learning disabilities or neurodivergent issues, may hinder youth from navigating and moderating their use of the dMH platform. The perceptions of the mental health professionals in our study are consistent with findings which show that certain conditions, as described by therapists, may not be a “good fit” with dMH and MBC.^72^ While dMH interventions are increasingly available, there is limited knowledge regarding the effectiveness of dMH for some serious mental health challenges, including psychosis and substance use disorders.^73^ There are aspects of certain mental health conditions, including depression, paranoia, and psychosis, that may make it difficult for an individual to engage with or trust digital technology.^74^ However, Birnbaum et al.'s research demonstrated that 35% of youth diagnosed with psychotic disorders used the Internet as their primary source of mental health information during their pathway to care.^75^ Among more stigmatized diagnoses, dMH may feel more acceptable for the youth as it can reduce barriers to accessing treatment and increase comfort with disclosure of symptoms.^34^ Given the broad literature that outlines both the potential of and concerns about dMH for certain mental health disorders, it is evident that more research is needed to explore the application of dMH with different types of mental health disorders in youth to determine their effectiveness and appropriateness.

Our study demonstrated that mental health participants are concerned that some youth will magnify their symptoms as a way to urgently access care or become overly identified with symptoms highlighted from engagement with MBC. Findings related to mental health professional perceptions of the potential negative consequences of MBC are mixed. On the one hand, dMH may unintentionally lead some youth to self-diagnose which may contribute to unnecessary anxiety, misinterpretation of one's experiences, and self-stigmatization.^76^ On the other hand, Rutter et al.’s study of over 3000 post-secondary students found that self-reported measures provide reliable information about symptoms and symptom severity.^77^ While our findings provide novel insights about professionals’ concern for the potential for youth to mis- or over-self-diagnose mental health disorders, the literature validates this concern but also draws upon the validity of self-reported recognition of symptoms and self-diagnosis. Through the use of dMH-enabled MBC, we believe there is an opportunity for mental health professionals to facilitate therapeutic discussions about youths’ lived experiences and the impact of symptoms and diagnoses on their lives to help collaboratively determine the right care.

Participants were also concerned about dMH suitability for youth with neurodevelopmental disorders, with a particular concern about youth with ASD, ADHD, and FASDs. In line with the participants in this study, youth with different types of diagnoses may have variable interest in technology, which is important to consider and explore.^78^ It is imperative that dMH interventions continue to be tailored to the needs of specific target users by adapting the content and interface to accommodate diverse psychosocial contexts and cognitive functioning.^79,80^ In this study, while participants preferred selectively using dMH only with youth whom they deemed appropriate, restricting dMH to youth with mild mental health symptoms may exclude many youth who may otherwise benefit greatly from dMH interventions. From an equity perspective, we strongly recommend mental health professionals empower youth to make their own decisions about using dMH regardless of their presentation and diagnosis.

Despite participants highlighting youth as yielding a strong interest in technological innovations, mental health care professionals in both SMHS and PCNs described a concern about youth readiness to use dMH, coupled with a lack of motivation and interest to complete measures among young people. There is some evidence to support that youth may be interested in participating in dMH, particularly the symptom measures, if the measures target changes that are important to them.^81^ As a result, MBC should use assessments that are sensitive to the goals and developmental stages of youth.^82,83^ While professionals may have the perspective that youth may not be interested or motivated, existing research demonstrates the opposite. One of the first places that young people tend to search for answers or support is the Internet, showcasing comfortability with the digital world.^84,85^ Further, research has shown that the youth may be more likely to engage with dMH tools for measurement, treatment, and ongoing support of their mental health challenges.^84^

By addressing the barriers noted by professionals as hindering youth's access to and use of dMH, we may enable the offering of dMH among a broader scope of youth. Reducing the perceived burdens of mental health professionals regarding their concerns about using dMH with youth experiencing moderate and serious mental health symptoms and acute mental health crises may ensure that more youth will be supported to use dMH-enabled MBC.^62^ The identification of mental health professionals’ perceptions of barriers and facilitators that may impact the use of dMH may contribute to the development of educational material to address misconceptions about who is and is not eligible and appropriate for dMH. Greater training may be required to equip mental health professionals to use dMH with diverse youth and families in different contexts. We provide greater detail on recommendations below in Table 3.

## Recommendations

### Strengths and limitations

There are several strengths and limitations to this study. An important strength of this study is the intentional involvement of youth research partners who participated in the analysis of focus group data collection, analysis, and manuscript writing. In addition to this, we recruited mental health care professionals from SMHS and PCNs in diverse rural, remote, and urban communities across Alberta. The province is large, and so being mindful of diverse geographical contexts was an important consideration. A final strength to highlight is the large sample size of 103 mental health professionals across the province which allowed for a range of perspectives. With respect to limitations, the presence of management in some focus groups may have impacted what was shared during a discussion. For some participants, pre-implementation of dMH-enabled MBC felt aspirational and hypothetical because they had not started using dMH yet. Therefore, participants may have been better able to speak to some of the facilitators and barriers after using the platform in the early and post-implementation phases of the project. There were also fewer youth-specific facilitators noted than barriers.

### Future research

There is a breadth of opportunities for future research directions. Future research could focus on eliciting more youth-focused facilitators and explore youth perspectives and experiences using dMH-enabled MBC tools. Mental health care professionals’ experiences and perspectives of using dMH-enabled MBC tools could also be further explored once they start using and engaging with digital MH tools. Future studies could also assess the use of dMH-enabled MBC in diverse service settings. The role of families and parents was an important theme that emerged all throughout the data. dMH implementation could be enhanced through research by doing co-design work with youth, families, and other professionals to culturally adapt dMH tools and options. In addition, research could assist in strengthening the evidence base by evaluating outcomes among diverse youth and dMH suitability.^86^ Lastly, more research is needed to identify the application of dMH with different types of mental health disorders.

## Conclusion

Within our study we sought to explore barriers and facilitators to implementation of a dMH platform in diverse service settings. The findings revealed that mental health professionals yield opinions and perceptions about barriers and facilitators related to youth and youth context to engage with dMH-enabled MBC tools. The findings reveal complexity in their concerns that may inadvertently point to misconceptions that should be addressed in implementation strategies. By identifying professionals’ perceptions, we may better understand how to work through potential barriers and facilitators in the implementation of dMH. These learnings can be applied to training and education development on how to support the youth from diverse contexts and with complex mental health issues to access and use dMH tools. Our hope is that this study may be beneficial to ensure mental health professionals feel supported and youth are made aware of all options available to them so that they can be included in making decisions about their mental health care journey.

## Supplemental Material

sj-docx-1-dhj-10.1177_20552076241253093 - Supplemental material for Perceptions of mental health providers of the barriers and facilitators of using and engaging youth in digital mental-health-enabled measurement based care Supplemental material, sj-docx-1-dhj-10.1177_20552076241253093 for Perceptions of mental health providers of the barriers and facilitators of using and engaging youth in digital mental-health-enabled measurement based care by E.M. Bassi, K.S. Bright, L.G. Norman, K. Pintson, S. Daniel, S. Sidhu, J. Gondziola, J. Bradley, M. Fersovitch, L. Stamp, K. Moskovic, H.M. LaMonica, F. Iorfino, T. Gaskell, S. Tomlinson, D.W. Johnson and G. Dimitropoulos in DIGITAL HEALTH
