# Supplementary material for: Perceptions of mental health providers of the barriers and facilitators of using and engaging youth in digital mental-health-enabled measurement based care
Source: Digit Health. 2024 May 7;10:20552076241253093. doi: 10.1177/20552076241253093 (PMC11080807; doi:10.1177/20552076241253093)
Supplement: sj-docx-1-dhj-10.1177_20552076241253093 - Supplemental material for Perceptions of mental health providers of the barriers and facilitators of using and engaging youth in digital mental-health-enabled measurement based care [file sj-docx-1-dhj-10.1177_20552076241253093.docx]

Qualitative Interview Guide

HEALTH CARE PROFESSIONALS

eMH Study

Community & Service Setting:

Date:

1. prep presentation

2. chat box

3. confirm consent

4. Housekeeping

- Chat okay
- Cameras on
- Diverse views
- Not Q&A
- Purpose of the research – learning; preliminary synthesis – help us help you with implementation in your setting; put all community findings together to write and publish papers about the implementation of eMH
- Consent and recording discussion

***RECORD***

**Focus Group Questions:**

1. Now that you have learned about the platform, what thoughts are coming up for you?

- Probe for both positive and negative feelings/thoughts coming up about the platform.

2. What is unique about your organization?

- Probe for unique aspects as a clinical team, including, who they serve.

3. What is unique about your community that will influence how the platform is used?

- Probe about geographic location, culture, Internet connectivity, and other contextual variables.

4. What is unique about the population of youth that you serve in your community?

- Who will have the greatest difficulty accessing services?

5. How might the platform help with improving access to services?

6. What should we do if we want this project to fail in your community?
